# Ff-nano, short functionalized nanorods derived from Ff (f1, fd, or M13) filamentous bacteriophage

**DOI:** 10.3389/fmicb.2015.00316

**Published:** 2015-04-20

**Authors:** Sadia Sattar, Nicholas J. Bennett, Wesley X. Wen, Jenness M. Guthrie, Len F. Blackwell, James F. Conway, Jasna Rakonjac

**Affiliations:** ^1^Institute of Fundamental Sciences, Massey UniversityPalmerston North, New Zealand; ^2^Science Haven Limited, Palmerston NorthNew Zealand; ^3^University of Pittsburgh School of MedicinePittsburgh, PA, USA

**Keywords:** phage display, filamentous phage, M13 phage, fd phage, f1 phage, lateral flow, nanorod, fibronectin-binding protein

## Abstract

F-specific filamentous phage of *Escherichia coli* (Ff: f1, M13, or fd) are long thin filaments (860 nm × 6 nm). They have been a major workhorse in display technologies and bionanotechnology; however, some applications are limited by the high length-to-diameter ratio of Ff. Furthermore, use of functionalized Ff outside of laboratory containment is in part hampered by the fact that they are genetically modified viruses. We have now developed a system for production and purification of very short functionalized Ff-phage-derived nanorods, named Ff-nano, that are only 50 nm in length. In contrast to standard Ff-derived vectors that replicate in *E. coli* and contain antibiotic-resistance genes, Ff-nano are protein-DNA complexes that cannot replicate on their own and do not contain any coding sequences. These nanorods show an increased resistance to heating at 70^∘^C in 1% SDS in comparison to the full-length Ff phage of the same coat composition. We demonstrate that functionalized Ff-nano particles are suitable for application as detection particles in sensitive and quantitative “dipstick” lateral flow diagnostic assay for human plasma fibronectin.

## Introduction

Filamentous bacteriophages are filament-like bacterial viruses (Day, 2011; Rakonjac et al., 2011). The F-pilus-specific filamentous phage of *Escherichia coli*, Ff (f1, M13, and fd), are resistant to heat, wide range of pH and ionic detergents, to which the tailed phage, such as λ, are sensitive (Branston et al., 2013). The Ff phage virions are 860 nm in length and 6 nm in diameter; they contain circular single-stranded DNA of ∼6400 nt (Day et al., 1988; Marvin et al., 2014). The virion is composed of five proteins. The major coat protein pVIII, that forms the shaft of the filament, is present in the virion in a few thousand copies. Two pairs of minor coat proteins, pIII/pVI and pVII/pIX, form two asymmetric ends of the filament. They are each present in the virion in up to five copies. Infection with filamentous phage is mediated by binding of a minor protein, pIII, to the primary and secondary receptors, the tip of the F-pilus and the TolQRA protein complex spanning the periplasm and the inner membrane, respectively. The primary receptor, however, is not essential for infection; in the presence of Ca^2+^ ions as many as 1% of *E. coli* cells in the culture can be infected with Ff (Russel et al., 1988). TolQRA, a conserved protein complex in Gram-negative bacteria, appears to be both an essential and universally required protein for filamentous phage infection, allowing what appears to be a low-efficiency broad-spectrum infection of Gram-negative bacteria by this group of phage (Heilpern and Waldor, 2000).

After entry into the host cytoplasm, the circular ssDNA genome [the positive (+) strand] of Ff replicates as an episome via a rolling-circle mechanism, one strand at a time. Replication of each strand requires specific sequences called negative (-) strand origin and positive (+) strand origin [(-) and (+) *ori*] and host proteins (Model and Russel, 1988; Rakonjac et al., 2011). In addition, replication from (+) origin requires phage-encoded protein pII (Asano et al., 1999). In addition to origin of replication, a *cis* sequence called packaging signal (PS) is required for targeting the (+) strand ssDNA to assembly machinery and assembly of the virions (Russel and Model, 1989). The (-) and (+) strand origins of replication, as well as the packaging signal, are together often referred to as the f1 origin of replication and are located within a ∼400 nt long intergenic sequence (IG; Supplementary Figure S1A). When inserted into plasmids, the IG (f1 *ori*) allows rolling circle replication in the presence of a helper phage, followed by packaging of the resulting ssDNA into Ff-derived particles; such vectors are known as phagemids. Some phagemid vectors are designed to express translational fusions with the Ff virion proteins and are used in phage display technology (Vieira and Messing, 1987; Barbas et al., 1991).

The virion proteins are all integrated into the inner membrane prior to assembly. The Ff virions are assembled by transfer of proteins from the inner membrane onto the ssDNA genome through a mechanistically and structurally poorly understood secretion-like process, mediated by phage-encoded trans-envelope secretion-assembly machinery (Russel and Model, 2006; Rakonjac et al., 2011). Whereas the diameter of the wild-type Ff virion is constant, the length depends on the length of packaged ssDNA. The shortest Ff-derived particles reported were 50 nm in length, containing circular ssDNA of 221 nt (Specthrie et al., 1992). They were produced by helper-phage-assisted replication from a specially constructed origin of replication, 303 nt in length (Supplementary Figure S1B), inserted into a plasmid. This 303-nt sequence comprises two (+) origins of replication, *ori1* and *ori2*, flanking a packaging signal. The *ori1* corresponds to the core (I) region of the (+) *ori* and serves as an initiator of replication. The extended (II) region of the (+) *ori* was not included in the *ori1*, in order to minimize the replicon size and therefore the particle length. Replication in the absence of region II of the (+) *ori* requires a helper phage containing *gII^IRI^* mutant (Dotto et al., 1984). The *ori2* is further shortened; it does not bind pII^IRI^ and cannot serve as an initiator; its role is to terminate replication of the (+) strand. In the presence of pII^IRI^, a segment between the two pII cut sites (TTCTTT↓AATA) in the two (+) origins is replicated and the resulting 221 nt ssDNA is ligated to form a circular ssDNA molecule. A PS inserted in between the two (+) origins allows assembly of this short circular ssDNA into 50 nm-long Ff-like particles (Specthrie et al., 1992).

The physical properties of Ff phage, coupled with their amenability to genetic engineering using recombinant DNA technology, have enabled their extensive use in modern biotechnology and nanotechnology. Ff is central to phage display, a combinatorial technology in which libraries of peptides, antibodies, or proteins are displayed on the virion surface, whilst the corresponding coding sequences are encapsulated inside the virions. This physical link between the displayed protein and its coding sequence allows affinity screening and enrichment of rare variants that bind to a ligand or a bait, from vast libraries of variants (Smith, 1991; Rebar and Pabo, 1994; Zwick et al., 1998; Bradbury and Marks, 2004). The Ff phage have more recently been used as nanoparticle-templates to display arrays of organic and inorganic molecules (Bernard and Francis, 2014) for applications ranging from tissue targeting (Souza et al., 2010) and drug delivery (Bar et al., 2008) to nanoelectrodes (Lee et al., 2009), light-harvesting (Dang et al., 2013) and diagnostic devices (Petrenko, 2008). Furthermore, the liquid crystalline properties of Ff have been exploited to assemble colloidal membranes and other structures (Gibaud et al., 2012) and for applications in tissue engineering (Chung et al., 2011) and colorimetric sensors (Oh et al., 2014).

The current applications of Ff phage could be expanded by manipulating the length of the particles, potentially resulting in nanomaterials of novel properties. Short rods may be preferred over the long filaments in some applications, such as diagnostic methods that use diffusion (lateral flow) of diagnostic particles through complex matrices. Furthermore, short particles lacking viral or antibiotic-resistance genes would lower regulatory hurdles and consumer concerns, allowing wider application outside of laboratory containment.

To expand the versatility and decrease the risks Ff-derived nanoparticle use, we have developed a system for high-efficiency production of short functionalized Ff-derived particles (50 nm × 6 nm) that we named “Ff-nano.” These particles do not carry any genes and cannot replicate inside a bacterial cell nor can they integrate into bacterial chromosome. We show that these short particles are more resistant to heating in the presence of ionic detergent SDS compared to the full-length phage. Furthermore we demonstrate that functionalized Ff-nano can be used as detector particles for a high-sensitivity dipstick assay detecting a test analyte, fibronectin, at a concentration of 0.35 femtomoles/μL (2 × 10^8^ molecules/μL).

## Materials and Methods

### Bacteria, Phage, and Growth Conditions

All *E. coli* strains used in this study are derivatives of laboratory strain K12. They are listed in **Table 1**. Strain K561 was used for growth of the helper phage R408 [f1 Δ*PS gIX(T30A) gII^IRI^(C143T) gtrxA2*; Russel et al., 1986]. Strains containing a *supD* mutation, K1030, and K2092 were used for growth of *gVIII^amber^* mutant phage R777 (R408, *gVIII^am25^*) or Rnano3 (R777, MCS in *gIII*) or R408-3 (R408, MCS in *gIII*). K2092 was obtained by co-transducing the *supD* mutation with the *zed508*::Tn10 (Tet^r^ marker) from strain K1030 into strain TG1 (Carter et al., 1985) using generalized transduction with P1 (Cm^r^ Clr-100; Sternberg and Maurer, 1991). The *zed508::Tn10* (Tet^r^) transductants were tested for plaque formation by phage R777 (*supD* is co-transduced with *zed508::Tn10* at a frequency of 80%). Strain K1030 containing pIV-expressing plasmid pPMR132 (Linderoth et al., 1997) was used for growth of phage R676 (f1, *ΔgIV, gVIII^am25^*; Feng et al., 1999).

**Table 1:** Bacterial strains.

Strains^a^	Genotype	Reference
TG1	*supE44 Δ(hsdM-mcrB)5* (rk^-^ mk^-^ McrB^-^)* thi Δ(lac-proAB)* F’ [*traD36 lacI^q^ Δ(lacZ)M15 proA^+^B^+^*]	Carter et al. (1985)
K561	*HfrC λ^+^ relA1 spoT1 T2^R^ (ompF627 fadL701) ΔlacZ lac1^q^*	The Rockefeller University collection
K1030	*HfrC λ*^-^* relA1 spoT1 T2^R^ (ompF627 fadL701) supD zed508::Tn10*	The Rockefeller University collection
K2092	*TG1 supD zed508::Tn10*	This work

*^a^All strains are derivatives of *Escherichia coli* K12.*

The cultures were propagated in Difco^TM^ 2xYT (Yeast Extract Tryptone) liquid medium [Becton-Dickinson (BD) and Company, USA] at 37°C with continuous shaking (200 rpm), or on 2xYT agar plates (1.2% Bacto-Agar, BD) at 37°C unless otherwise stated. The medium was supplemented with suitable antibiotics where required, sourced from Sigma–Aldrich and Goldbio (USA). Antibiotics were used at the following concentrations: Ampicillin (Amp) 100 μg/mL, Kanamycin (Km) 30 μg/mL, Chloramphenicol (Cm) 25 μg/mL, and Tetracycline (Tet) 15 μg/mL unless otherwise indicated.

### Construction of Phage and Plasmids

General molecular biology techniques for cloning, PCR amplifications and sequencing were carried out as described in (Sambrook and Russell, 2001). Molecular biology reagents were sourced from New England Biolabs Inc. (USA), Roche Molecular Biochemicals (Germany), Life Technologies Inc. (USA), and Takara (Japan). Oligonucleotides used in cloning, sequencing and PCR reactions were manufactured by Life Technologies Inc. and Integrated DNA Technologies Inc. (USA). DNA sequencing was carried out at the Massey University Genome Services, Institute of Fundamental Sciences, Palmerston North, New Zealand. The small and large scale plasmid and phage closed-circular double-stranded (RF) DNA was prepared using High Pure Plasmid Isolation Mini and Midiprep kits (Life Technologies; Roche Molecular Biochemicals) according to the manufacturers’ instructions.

Construction of the recombinant phage and the plasmid pNJB7 is described in Supplementary Methods and Supplementary Figure S2. Briefly, helper phage R777 was constructed from phage R408 and R676 (Feng et al., 1999), by combining the R408 origin of replication and *gII^IRI^* with *gVIII^am25^* of R676.

The helper/vector phage Rnano3 was constructed by combining the *gVIII^am25^* mutation with a 45-nt multiple cloning site (MCS) identical to that of the phage display vector pHEN2 (Marks et al., 1991) in the R408 background. Another helper/vector, containing the identical MCS, but wild-type *gVIII*, named R408-3, was constructed in parallel.

Recombinant helper/vector Rnano3FnB was constructed by inserting coding sequence of fibronectin binding (FnB) domain from *Streptococcus pyogenes* (Rakonjac et al., 1995) into the MCS of Rnano3.

The Ff-nano-producing plasmid pNJB7 was constructed by inserting the Ff-nano origin of replication amplified from plasmid pLS7 (Specthrie et al., 1992) into the high-copy-number vector pCR4-TOPO (Life Technologies).

### Phage Growth

All helper-vector phage stocks were prepared initially from a single plaque using a plate method. Briefly, phage were extracted from a plaque into 1 mL of 2xYT by slow rotation at room temperature for 1 h, filtered through a 0.45 μm-pore filter to remove the *E. coli* cells and titrated on an appropriate strain. To make a plate stock, 10^5^-10^7^ phage from the dissolved plaque were mixed with a culture of an appropriate host in 2xYT soft agar, plated, and incubated overnight. The phage were extracted into 5 mL of 2xYT overlaid onto the surface of the lawn, followed by slow shaking for 1 h at room temperature. The 2xYT containing extracted phage was collected and filtered to remove *E. coli*. Titers of the plate stocks were typically ∼10^11^ per mL. These plate stocks were then used for preparation of larger-volume stocks using a standard single-round infection of exponentially growing liquid cultures at a multiplicity of infection (m.o.i.) of 50 phage per bacterium.

### Purification of the Ff-nano Particles

An exponentially growing culture (2–8 L) of K1030 carrying pNJB7 (Ff-nano-producing plasmid) was infected with appropriate helper phage (R777, Rnano3, or Rnano3FnB) at an m.o.i. of 50 and incubated at 37°C without shaking for 20 min, to allow infection. Incubation was continued overnight at 37°C with shaking at 200 rpm. The subsequent day, cells were pelleted (7000 × *g* for 20 min at 4°C) and the supernatant containing the Ff-nano and full-length helper phage was subjected to differential two-step polyethylene glycol (PEG) precipitation, to separate the two types of particles from each other, as described (Specthrie et al., 1992). Briefly, in the first precipitation, culture supernatant was subjected to 2.5% w/v PEG8000/0.5 M NaCl and incubated overnight at 4°C to precipitate the full-length helper phage. Precipitated full-length helper phage were collected by centrifugation at 16,500 × *g* for 45 min at 4°C. Supernatant containing the Ff-nano particles was transferred to sterile containers, whereas the full-length phage pellet was suspended in TBS (50 mM Tris, 150 mM NaCl, pH 7.0). In the second precipitation, solid PEG8000 was added to the supernatant, to a final concentration of 15%, and incubated overnight at 4°C. The following day the Ff-nano-enriched precipitate was collected by centrifugation (at 16,500 × *g* for 45 min at 4°C) and the pellet was resuspended in TBS. The full-length phage and Ff-nano fractions (resuspended pellets from 2.5 and 15% PEG, respectively) were further subjected to successive detergent treatments and precipitations to decrease contamination with fragments of inner and outer membranes [Sarkosyl (1% w/v) and Triton X-100 (0.1% v/v)]. Full length helper phage were recovered after each detergent treatment by precipitation with 2.5% PEG and Ff-nano with 15% PEG as described above, except that precipitations were carried out at room temperature to decrease co-precipitation of detergents with the particles.

Ff-nano particles were further purified away from the remaining full-length helper phage based on the large difference in size. Native agarose gel electrophoresis was carried out as described (Nelson et al., 1981; Rakonjac and Model, 1998). Preparative native agarose gel electrophoresis was carried out on 0.8% agarose gels. Upon completion of electrophoresis, the agarose gel slabs containing the bands corresponding to full-length or Ff-nano particles were separately cut out and transferred to dialysis tubes (Novagen, D-tube dialyzer Maxi, MWCO 12–14 kDa) containing 500 μL of sterile 1 × TAE buffer (40 mM Tris-acetate buffer, pH 8.3, 1 mM EDTA). These tubes were then placed into an electrophoresis chamber in sterile 1 × TAE buffer and electrophoresed overnight at 0.5 V/cm at 4°C. After removing the gel slices from tubes, the phage particles (full-length or Ff-nano) were precipitated overnight at 4°C using 2.5 or 15% PEG, respectively, in 0.5 M NaCl. The precipitate was dissolved in an appropriate amount of 1 × PBS (137 mM NaCl, 2.7 mm KCl, 10 mM Na_2_HPO_4_, 1.8 mM KH_2_PO_4_, and stored at -80°C until further use.

### Quantification of Ff-derived Particles

Phage R777, Rnano3, and Rnano3FnB (containing *gVIII^am25^* mutation) were titrated on *E. coli supD* strains K1030 or K2092 to determine the number of plaque-forming units per milliliter, using standard phage plating, and titration methods. To quantify non-infectious Ff-nano, the particles were disassembled in SDS-containing buffer (1% SDS, 1 × TAE, 5% glycerol, 0.25% bromophenol blue) at 100°C for 5 min and subjected to electrophoresis in 1.2% agarose gel in 1 × TAE buffer. After electrophoresis, the ssDNA released from disassembled Ff-derived particles was stained with ethidium bromide (Supplementary Figure S3) and quantified by densitometry (Rakonjac and Model, 1998). The amount of ssDNA in any band is not linearly proportional to the intensity of fluorescence, therefore each gel contained a set of twofold dilutions of purified full length f1 ssDNA that were used for calibration (Supplementary Figure S3). Gels were photographed by a CCD camera (Biorad, USA). Image Gage V4.0 and Microsoft Excel were used for densitometry and calculations, respectively. A second-order polynomial function was used to fit the standard curve. Amounts of ssDNA were converted to the genome equivalents based on the molecular weight of the ssDNA genome. The molecular weight of the Ff-nano ssDNA genome was in turn calculated from its length (221 nt) and nucleotide composition (derived from the DNA sequence).

Native agarose gel electrophoresis was used to separate the intact full-length (helper) phage from Ff-nano particles, for analysis and purification. The bands containing intact particles were detected in agarose gels after the *in situ* stripping the virion proteins off the ssDNA by soaking the gel in 0.2 M NaOH for 1 h, followed by neutralization with 0.45 M Tris pH 7.0 and staining with ethidium bromide (Supplementary Figure S4).

### Microscopy

The phage samples for non-cryo TEM analysis were adsorbed onto glow-discharged carbon-coated grids, negatively stained with a 1% uranyl acetate solution, and examined on a Phillips CM-10 microscope at the Manawatu Microscopy and Imaging Centre (MMIC, Institute of Fundamental Sciences, Massey University).

Cryo-negative staining was performed at the University of Pittsburgh, Pittsburgh, PA, USA, on an FEI (Hillsboro, OR) Tecnai F20 microscope equipped with a Gatan (Pleasanton, CA, USA) 626 cryoholder and operated at 200 kV and nominal magnification of 50,000×. Briefly, 3 μL of sample were pipetted onto a holey carbon/copper grid that had been briefly glow-discharged. The grid was then placed sample-down onto a 100 μL droplet of 16% ammonium molybdate (in the pH range 7.0–8.0) and floated for 60 s, following the cryo-negative staining procedure (Adrian et al., 1998). The grid was then removed, blotted and plunge-frozen into liquid ethane using an FEI Vitrobot Mk III. Images were collected using standard low-dose techniques on a Gatan Ultrascan 4000 CCD camera with post-column magnification of 1.33×, yielding a pixel size at the sample of 2.3 Å.

### Phage ELISA Assay

Phage Enzyme Linked Immunosorbent Assay (ELISA Harlow and Lane, 1999) was carried out in the 96-well plates (Nunc-Immuno MaxySorpTM, Denmark). The wells were coated overnight at 4°C with 100 μL of human plasma fibronectin (Sigma–Aldrich, USA) at 20 μg/mL in 100 mM sodium bicarbonate buffer, pH 9.5. All subsequent steps were carried out at room temperature. The wells were washed once with 300 μL of PBS containing 0.05% Tween 20 (PBST) and blocked with 250 μL of 2% (w/v) Bovine Serum Albumin (BSA) in PBS for 2 h. After washing wells three times with 300 μL of PBST, Rnano3FnB full-length phage or Ff-nano particles (1 × 10^8^) in 100 μL of PBS were added to the wells. Negative controls (buffer and protein) were PBS and 2% BSA in PBS, respectively, whereas negative phage controls were the full-length phage and the Ff-nano particles derived from “empty” vector/helper Rnano3 (and therefore not displaying FnB). The plates were then incubated for 2 h. The unbound particles were removed by extensive washing with PBST (300 μL, seven times). To detect phage particles bound to fibronectin in the wells, 100 μL of rabbit anti-pVIII (polyclonal antibody to M13, fd, and f1, Progen Biotechnik; Germany) was added at the concentration of 0.1 μg/mL in 1× PBS and incubated for 1 h. The wells were then washed five times with 300 μL PBST buffer. Next, 100 μL of secondary HRP-conjugated anti rabbit antibody was added to all wells at a dilution of 1:2000 and then washed with 300 μL PBST seven times. The HRP bound to the plate was detected with 1-StepTM Turbo TMB-ELISA reagent (Thermo Scientific). The enzyme reaction was stopped by adding 25 μL of 0.5 M H_2_SO_4_. The signal was quantified by measuring absorbance at 450 nm.

### Labeling of Ff-derived Particles Using Fluorescein Isothiocyanate

Fresh Fluorescein IsoThioCyanate (FITC) solution (1 mg/mL) was prepared in 1 M NaCO_3_/NaHCO_3_ (pH 9.0) buffer. Ff-nano (0.5 × 10^13^ in 500 μL) was precipitated using 15% PEG/0.5 M NaCl and the same amount of the full-length phage with 2.5% PEG/0.5 M NaCl. The precipitate was dissolved in 200 μL of 1 M NaCO_3_/NaHCO_3_ (pH 9.0) buffer. The FITC solution (50 μL) was added to the suspension of precipitated Ff-nano or full-length phage and the reaction mixture was rotated in the dark for 1 h at room temperature. The reaction was stopped by adding 10 μL of the NH_4_Cl and the particles were purified by a series of two PEG8000 precipitations using the concentrations appropriate for Ff-nano (15%) and full-length phage (2.5%). The pellet obtained after the second PEG8000 precipitation was suspended in 100 μL of 1× PBS and stored at 4°C in the dark until further use.

### Dipstick Assays

Hi-Flow Plus membrane cards were used to make the dipsticks (Millipore Corporation). These cards contain nitrocellulose membranes cast on a 2 mil (0.001 inch; USA) polyester film backing. The reagents for the test and control lines were applied to the membrane using a mechanical dispenser. Collagen I (Sigma; 1 μg/μL in 0.25% acetic acid;) was dispensed at the test line, whereas undiluted mouse anti-pVIII antibody or anti-FITC antibody (0.5 to 1 μg/μL) was dispensed at the control line. The protein-loaded cards were allowed to dry at 37°C for 1 h and were subsequently cut into 0.5 cm × 2.5 cm size dipsticks using an automatic card-cutting tool (BIODOT membrane cutter; SM5000; sheet slitter). The dipsticks were stored in zip-lock sealed bags at room temperature in a dark place until use. The membrane has a flow rate of 46 s/2.5 cm (total length of the dipstick).

The distal end of protein-loaded dipstick was dipped into the 50 μL mixture of analyte (fibronectin, 5 μg/50 μL for initial detection and serial twofold dilutions starting from 0.5 μg/50 μL for quantitative detection) and the detection particles that was pre-incubated for 30 min. Full-length phage were used at 1 × 10^10^ per assay, and Ff-nano (or Ff-nanoFnB) particles at 1 × 10^11^ per assay. Dipsticks were allowed to stand in solution for 30 min and dried for 1 h at 37°C. The FITC-labeled particles on the dipsticks were detected using a phosphoimager (Fuji, Japan). Unlabeled particles were visualized by immune-blotting using rabbit pVIII-specific antibody (0.66 μg/mL; Progen Biotech) for 1 h at room temperature, followed by five washes of 5 min each with PBST. Rabbit IgG-specific antibody conjugated to alkaline phosphatase (Sigma, USA) was used as the secondary antibody. The dipsticks were washed again five times with PBST, and developed using Nitro Blue Tetrazolium (NBT) and 5-Bromo-4-Chloro-3-Indolyl Phosphatase (BCIP) in alkaline buffer (Blake et al., 1984). Densitometric analysis of the signal at the control and test lines in quantitative assays was performed using ImageJ software (Schneider et al., 2012).

## Results

### The Ff-nano Production and Purification System

**FIGURE 1:**
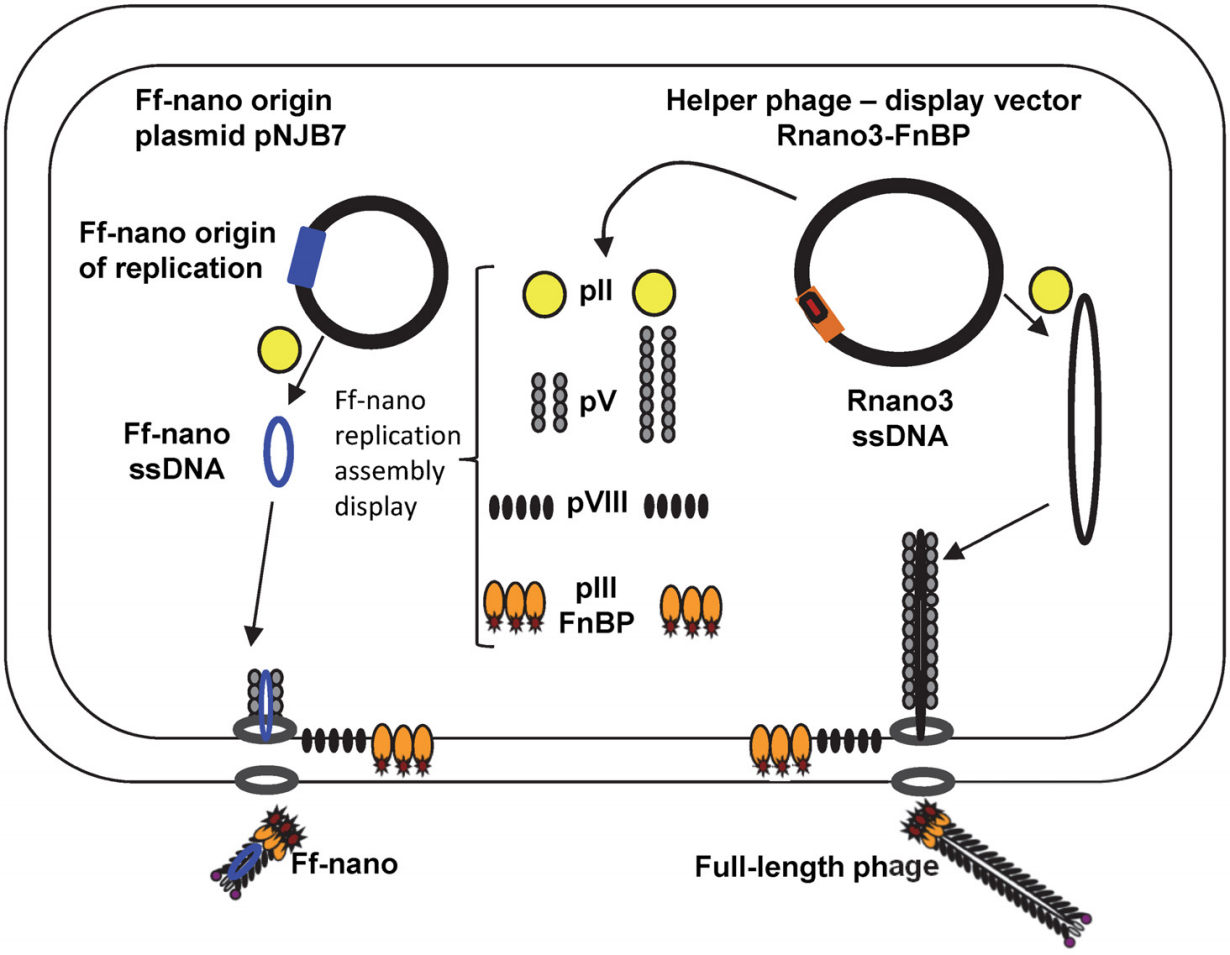
**The system for production of functionalized Ff-nano.** *Escherichia coli* cells containing the Ff-nano production plasmid pNJB7 were infected with the helper phage Rnano3FnB containing the coding sequence of a “probe” or “detector” protein fused to pIII. Upon infection, pII from the helper phage induces positive strand replication from the pNJB7 Ff-nano origin of replication and also provides all other phage proteins and assembly machinery for production of the Ff-nano particles. All five copies of pIII are fusions to the probe (only three copies of pIII fusions are shown).

**FIGURE 2:**
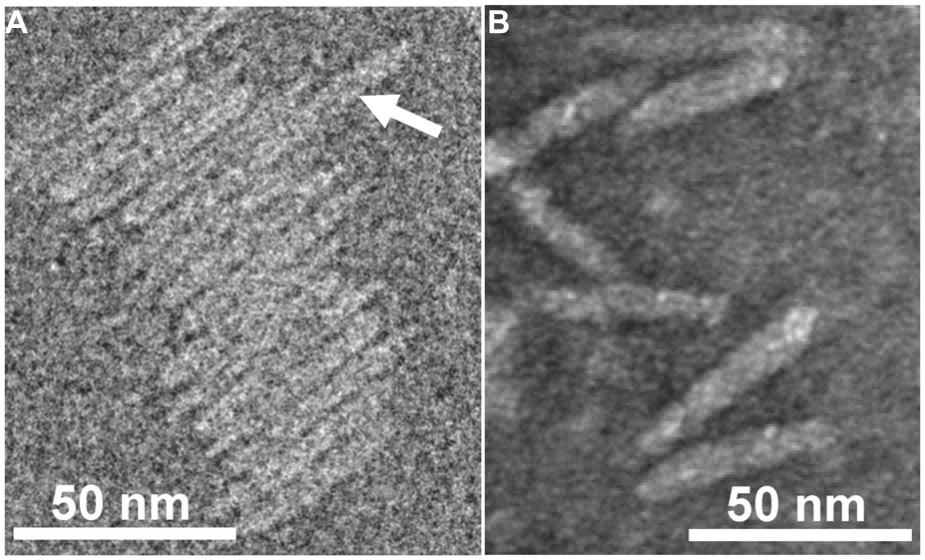
**Transmission electron micrographs (TEM) of Ff-nano particles. (A)** Cryo-negative TEM of particles obtained using the R777 helper phage. **(B)** Negative-stain TEM of particles obtained using the Rnano3 helper/vector. Arrow in **(A)** points to a double-length particle. Samples were prepared as described in Section “Materials and Methods.”

In this work we have constructed an Ff-derived phage display system for production of functionalized nanorods (50 nm × 6 nm) we named Ff-nano (**Figure 1**). In the first stage, we modified a setup for production of short phage originally published by Specthrie et al. (1992) to increase the amount (per cell) of the 221-nt circular ssDNA available as a template for assembly of the short particles. This was achieved by inserting the 303-nt short-phage (microphage or Ff-nano) origin of replication (Supplementary Figure S1) into the high-copy-number plasmid pCR4-TOPO (in contrast to the low-copy-number plasmid pBR322 used in the original system), to obtain plasmid pNJB7. Furthermore, a new helper phage, named Rnano3, was constructed. The helper phage Rnano3, in contrast to the original helper phage R474 used for short phage production (Specthrie et al., 1992), is not only a helper but also a phage display vector. Rnano3 was designed for display of proteins as fusions with phage protein pIII that is present in up to five copies at one end of the Ff-derived particles. Infection of cells containing plasmid pNJB7 with the helper phage/vector Rnano3 resulted in production of the 50 nm × 6 nm particles (Ff-nano or microphage; **Figure 2**) and full-length phage.

The 50-nm-particle production was originally reported to be improved in the presence of helper phage that produces decreased amount of the major coat protein pVIII. Decreased amount of pVIII presumably favors initiation and termination of assembly over elongation of particles, thereby decreasing the co-packaging of short 221 nt ssDNA with the helper phage DNA into the full-length particles and increasing production of short particles (Specthrie et al., 1992). In contrast to R474 helper used previously for the short phage production, which has a mutation lowering transcription of not only *gVIII* but also in *gIX* (Russel and Model, 1989; Specthrie et al., 1992), the Rnano3 helper phage/vector that we constructed produces decreased amount of pVIII without affecting *gIX* expression. This specific effect on pVIII is achieved by using a suppressed *gVIII^am25^* mutation, which contains a TAG stop codon in the *gVIII* sequence at codon 25 (replacing the GAG codon for Glu). When the Rnano3 phage infects a *supD* strain, the TAG codon is translated into Glu or Ser (Rogers and Soll, 1988). The efficiency of the *gVIII^am25^* mutant translation in the *supD* strains (K1030 and K2092) is <50% that of the wild-type, resulting in a lower number of pVIII copies produced per cell than in an infection with phage containing a wild-type *gVIII*. Besides the helper/vector with decreased amount of pVIII, we also constructed and examined helper/vector R408-3 that is identical to Rnano3 apart from having a wild-type *gVIII*. Rnano3 and R408-3 were both found to support production of the Ff-nano particles (Supplementary Figure S5). As predicted, co-packaging of the 221-nt ssDNA derived from the Ff-nano origin of replication with the full-length phage genome appeared to be increased in the R408-3 full-length phage fraction after differential PEG precipitation, relative to that of Rnano3 (*gVIII^am25^* mutant), however, the production of Ff-nano particles was also increased (Supplementary Figure S5).

Ff-nano particles were partially purified away from the full-length helper phage by differential PEG precipitation (see Materials and Methods). The full-length helper phage was first precipitated out of the culture supernatant in 2.5% PEG8000, 0.5 M NaCl, followed by increase in PEG8000 to 15% to precipitate Ff-nano (Specthrie et al., 1992). However, this method resulted in 0.7% full-length phage still remaining in the Ff-nano fraction (**Table 2**). We used native agarose gel electrophoresis to separate the short particles from the full-length phage following the differential PEG precipitation (Supplementary Figure S4). Preparative agarose gel electrophoresis is simple, fast, effective, and less expensive than the size fractionation by Sepharose CL2B columns used by Specthrie et al. (1992). The band containing Ff-nano was excised and the particles were extracted by electroelution (see Materials and Methods for details of purification). The native preparative agarose gel electrophoresis purification step decreased the full-length helper phage frequency in the final purified sample by a factor of 1400, down to 5.0 × 10^-6^ relative to Ff-nano (**Table 2**). This method was also relatively efficient in recovery of the Ff-nano (31% of the input; **Table 2**).

**Table 2:** Purification of the Ff-nano by native agarose gel electrophoresis and electroelution.

	Ff-nano (total number^c^)	Recovery of the Ff-nano^d^	Helper phage (total number^e^)	Helper: Ff-nano ratio^f^	Fold decrease of helper: Ff nano ratio^g^
Input^a^	6.4 × 10^14^	N/A (input)	4.5 × 10^12^	7.0 × 10^-3^	N/A (input)
Electro-purified Ff-nano^b^	2.0 × 10^14^	31%	9.9 × 10^8^	5.0 × 10^-6^	1400

^a^Starting material loaded onto the preparative gel corresponds to the Ff-nano-enriched lysate obtained by differential PEG precipitation.

^b^Ff-nano-band was excised from the agarose gel, extracted by electroelution, purified and concentrated (see Materials and Methods).

^c^Determined by densitometry (see Materials and Methods).

^d^Ratio of the Ff-nano number in the electro-purified sample to the amount in the input.

^e^Determined by titration.

^f^Determined by dividing the total number of full-length helper phage with the total number of the Ff-nano particles.

^g^Fold decrease of the helper to Ff-nano ratio (“helper**:** Ff-nano ratio”) was calculated by dividing the “helper**:** Ff-nano ratio” in the input with the “helper**:** Ff-nano ratio” in the electro-purified sample (data column four; row one divided by the row two value).

### Physical Properties of the Ff-nano

#### Morphology

Purified Ff-nano were negatively stained and visualized by cryo-electron and transmission electron microscopy (**Figure 2**). Dimensions of the Ff-nano are 50 nm by 6 nm, matching those reported in Specthrie et al. (1992). The Ff-nano particle termini appear asymmetric, one pointy, and one blunt, as reported for the Ff phage (Gray et al., 1979). In the cryo-negative micrographs Ff-nano formed sheets composed of individual Ff-nano aligned in alternating orientations. In addition, in a larger field, about 3.5% (6 out of 172) double-length Ff-nano particles could be observed (Supplementary Figure S6). This is consistent with observations of full-length filamentous phage, which, depending on the genotype, contain some proportion of particles that are longer than the majority by a factor of two or multiple lengths of the virion, and containing two or more sequentially packaged genomes (Rakonjac and Model, 1998). We note that the Ff-nano particles visualized in the electron micrographs do not have any signs of the extra balls of density that are sometimes observed attached to the pIII end of the filament and correspond to free-moving N1N2 domains of pIII (Gray et al., 1979).

#### Stability to Heating in the Presence of Sodium Dodecyl Sulfate

**FIGURE 3:**
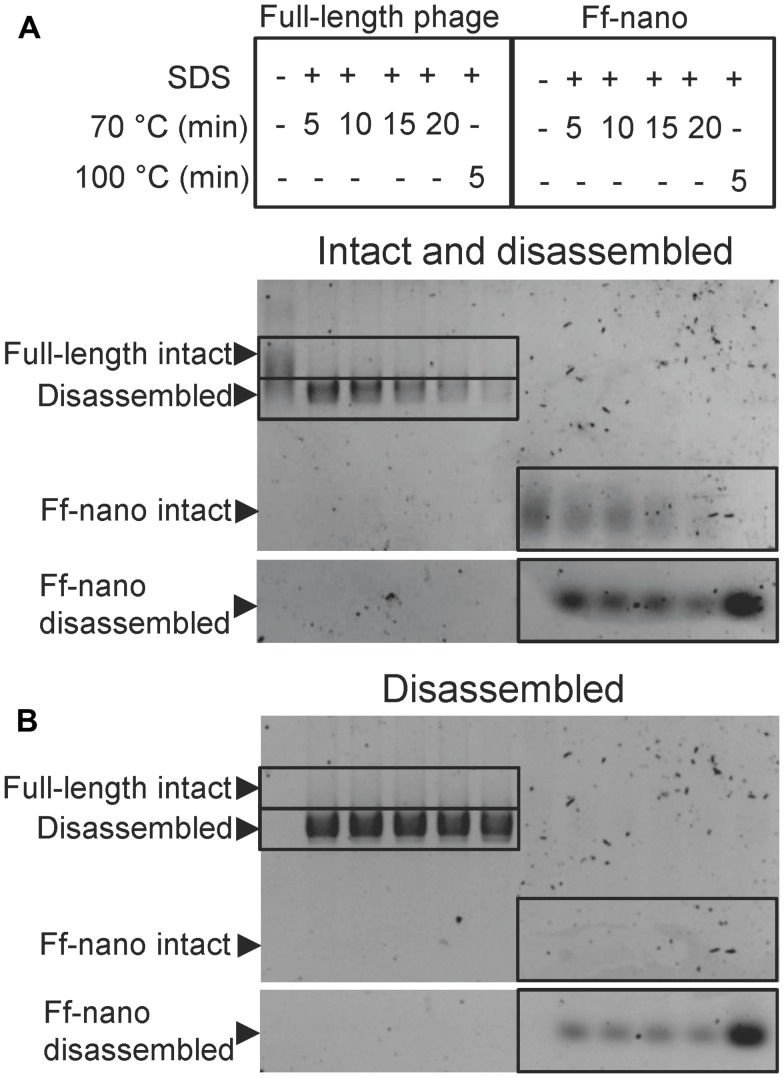
**Ff-nano resistance to heating in SDS. (A)** Intact (undamaged) particles and free ssDNA released from particles by the heat/SDS-treatment. Free ssDNA was visualized by staining the gel with ethidium bromide. Bands corresponding to the intact particles were visualized after soaking the gel in 0.4 M NaOH (see Materials and Methods for details). Gel sections containing the bands corresponding to the free ssDNA and intact full-length and Ff-nano particles are boxed. **(B)** Free ssDNA only, released by the heat/SDS treatment (visualized by direct ethidium bromide staining of the gel prior to NaOH treatment).

In the course of Ff-nano analyses, we observed that the standard protocol for Ff phage *in vitro* disassembly, heating in a buffer containing 1% (34 mM) ionic detergent sodium dodecyl sulfate (SDS) for 5 min at 70°C, was not efficient in releasing ssDNA from the Ff-nano particles (data not shown). This indicated that the Ff-nano particles could be more stable to heat/SDS treatment than the full length helper phage, even though both are assembled within the same *E. coli* cell. To test this hypothesis, a time-course experiment of heat exposure was used to monitor disassembly of full-length helper phage and Ff-nano isolated from the same culture using differential PEG precipitation and preparative agarose electrophoresis, as described in the previous section. Approximately 2 × 10^12^ Ff-nano or 1 × 10^11^ full-length phage were heated at 70°C in the presence of 1% SDS for a period from 5 to 20 min; one sample was also incubated at 100°C for 5 min. Disassembly of the helper (full-length) phage and Ff-nano particles was monitored by agarose gel electrophoresis (**Figure 3**). Released ssDNA was directly visualized by staining with ethidium bromide (**Figure 3B**), while the ssDNA that remained encapsidated inside the intact Ff-derived particles (resistant to heat/SDS treatment) was not detectable by direct staining. In order to visualize the SDS-resistant intact particle bands after electrophoresis, the coat proteins were dissociated from ssDNA *in situ* by soaking the gel in an alkaline buffer (0.4 M NaOH), followed by neutralization and re-staining of the gel by ethidium bromide (**Figure 3A**).

When the full-length (helper) phage virions were analyzed, the untreated samples (without SDS or heating) did not contain any free ssDNA. All full-length phage were therefore intact (all ssDNA was contained within the virion; **Figure 3**; compare the corresponding untreated sample lanes in gels A and B). In the presence of 1% SDS, at the first time-point (5 min) of incubation at 70°C, however, all ssDNA was in the free form and none in the intact phage particles, hence the vast majority of the full-length phage particles were sensitive to these conditions (**Figure 3A**). In contrast, when the Ff-nano particles were subjected to the same treatment (1% SDS at 70°C), a large proportion of the intact Ff-nano particles were detected, with some free ssDNA observed at all time-points (**Figure 3**, compare the 70°C-treated sample lanes in A vs. B). The amount of intact Ff-nano particles decreased gradually at 70°C between the 5 and 20 min time points, but nearly half stayed intact throughout the incubation. The intact Ff-nano were completely eliminated only after incubation at 100°C, confirming that only at this higher temperature is SDS able to disassemble all Ff-nano particles in the sample (**Figure 3**, compare lane 12 in A vs. B). This experiment demonstrates that the Ff-nano particles have superior resistance to heating in the presence of ionic detergent SDS in comparison to the full-length phage, requiring higher temperature (100 vs. 70°C) for dissociation of most particles.

### Functionalization of the Ff-nano

To test the potential of the Ff-nano-production system to assemble functionalized particles that can be used for display of foreign proteins, a fusion to pIII was constructed in the helper/vector Rnano3. The displayed peptide was the FnB domain from serum opacity factor (Sof), a surface protein of *S. pyogenes* (Rakonjac et al., 1995). The Sof FnB domain is composed of three repeats of a bacterial FnB motif (PF02986) that each binds to the N-terminal domain of Fibronectin (Fn) with a low nanomolar dissociation constant (Rakonjac et al., 1995; Schwarz-Linek et al., 2006). The high affinity of FnB for the Fn makes it a good candidate as a detector molecule and FnB-Fn combination is a good detector-analyte pair to investigate the use of Ff-nano as a display particle and for its applicability to lateral flow dipstick diagnostic devices.

**FIGURE 4:**
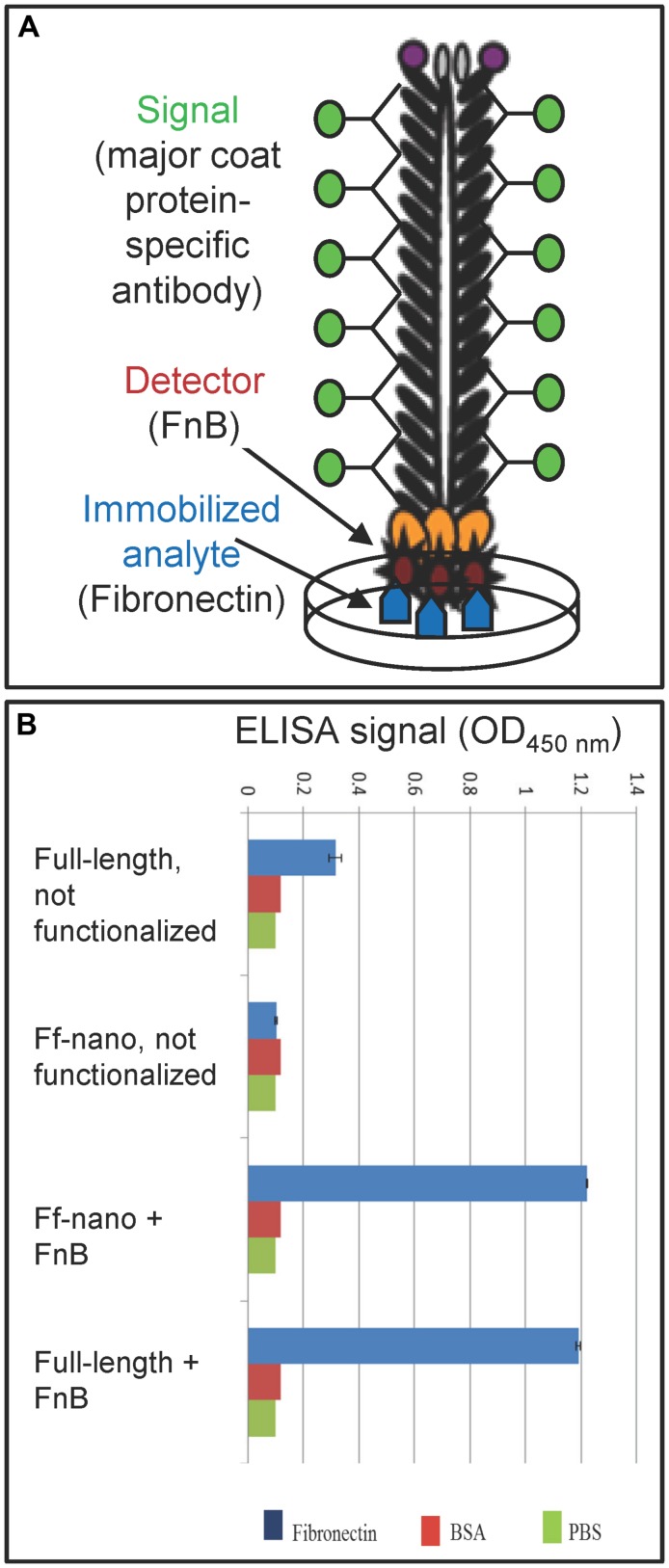
**Confirmation of Ff-nano-FnB display by phage ELISA assay. (A)** Schematic representation of the fibronectin-binding phage ELISA assay. **(B)** Assay result. Fibronectin was immobilized on a microtiter plate at a saturating concentration of 40 ng/μL (2 μg per well of a 96-well plate), whereas control wells for each assay were coated with PBS alone or BSA (1%) in PBS. The wells were exposed to (1 × 10^8^) Ff-nanoFnB or full-length phage Rnano3FnB that both displayed FnB as a pIII fusion, or control particles Ff-nano and Rnano3 that did not display FnB. After washing of the wells, bound Ff-nano or full-length phage were detected using a primary antibody to the major coat protein, then the secondary HRP-conjugated antibody, followed by detection of HRP through an enzymatic reaction according to the standard ELISA protocols. Data are presented as an average of three measurements. Error bars show standard deviation.

The functionalized helper/vector encoding the FnB-gIII fusion was named Rnano3FnB. When *E. coli* containing the Ff-nano-origin plasmid (pNJB7) was infected with Rnano3FnB, the Ff-nano particles displaying FnB (named Ff-nanoFnB) were produced together with the full-length helper phage (**Figure 1**). The helper (full-length) Rnano3FnB phage and the Ff-nanoFnB particles obtained using this helper were separated by differential PEG precipitation and each of the long and the short phage were further purified using native agarose gel electrophoresis followed by electroelution, as described in the previous section. Phage ELISA was performed to confirm that the FnB domain was displayed (schematically represented in **Figure 4A**). Rnano3FnB full-length and Ff-nanoFnB particles exhibited binding to immobilized fibronectin as indicated by strong ELISA signal detected for fibronectin-coated wells incubated with these phage particles (OD_450_ = 1.2 with 10^8^ particles per well in the presence of 40 ng/μL of Fn). No signal over background levels (OD_450_ = 0.1; intrinsic to the phage ELISA) was detected in control wells incubated with Ff-nano particles that did not display FnB, derived from the infection with vector Rnano3 (without the FnB domain) nor in the wells coated with BSA only (from which fibronectin was omitted; **Figure 4B**), apart from the full-length Rnano3 particles (not displaying FnB) which gave a low signal OD_450_ = 0.3 in the presence of Fn. In conclusion, Ff-nanoFnB particles detected Fn and were hence displaying FnB. All Ff-nano and Ff-nanoFnB samples used in the assays were imaged by TEM to confirm the purity and morphology of the particles (data not shown).

### Development of a Lateral Flow Assay for Fibronectin Using Ff-nano

#### Dipstick Assays Using Ff-nano

**FIGURE 5:**
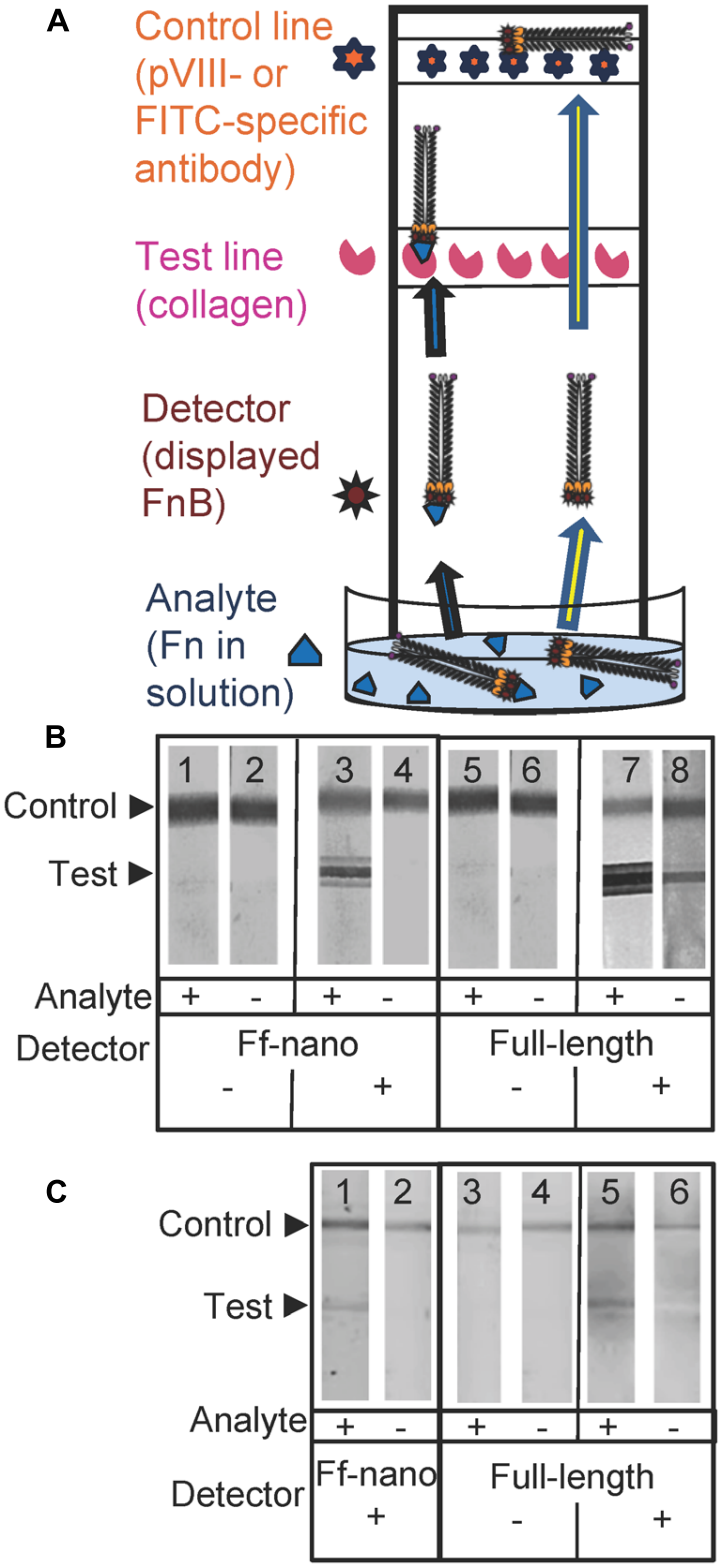
**Fibronectin dipstick assay using Ff-nanoFnB. (A)** Schematic representation of a lateral-flow dipstick assay; Fn-detection dipstick assay using: **(B)** unlabeled; **(C)** FITC-labeled particles. Each assay (50 μL) contained 1 × 10^10^ full-length Rnano3 (Rnano3FnB) or 1 × 10^11^ Ff-nano (or Ff-nanoFnB) particles and 1 μg of Fn. The assay was performed and the unlabeled or FITC-labeled particles were detected as described in Section “Materials and Methods.” The test line, printed with collagen solution, appears as a triple band when the signal is high. This is due to secondary lines flanking the main line that form during printing of collagen on the card. The triple banding during printing is caused by the acidity of the solution, necessary to keep the collagen soluble (0.25% acetic acid).

To investigate the detection of fibronectin (the analyte) in solution using the Ff-nano displaying FnB domains as the detector (probe) in lateral flow devices, a simple dipstick assay was designed and tested (see **Figure 5A** for a schematic representation of the Fn-detection dipstick assay). The dipsticks used in this assay contained human type I collagen at the test line. Collagen binds fibronectin with high affinity (Engvall et al., 1981).

The assays were carried out using particles that were either unlabeled (**Figure 5B**), or FITC-conjugated (**Figure 5C**). The unlabeled particles were captured on the control line using mouse pVIII-specific antibodies and detected on the dipstick, after the assay was completed, using rabbit pVIII-specific antibody. The FITC-labeled phage were captured on the control line using FITC-specific antibodies and detected on the dipstick using a phosphoimager.

As expected, the Ff-nanoFnB particles and full-length phage displaying FnB (Rnano3FnB) showed binding to collagen at the test line in the presence of fibronectin [**Figure 5B** (sticks 3 and 7); **Figure 5C** (sticks 1 and 5)]. The Ff-nanoFnB particles gave no background in the absence of Fn (analyte), whereas the full-length FnB-displaying phage showed some unspecific binding to the collagen test line (**Figure 5B**, dipstick 8 and **Figure 5C**, dipstick 6) in the absence of Fn. The Ff-nano and full-length particles that did not display FnB did not bind to the test line, indicating the lack of unspecific binding of phage to collagen [**Figure 5B** (sticks 1, 2, 5, and 6); **Figure 5C** (sticks 3, 4)].

#### Quantification of Fibronectin Using Dipstick Assay

To estimate the lower limit of detection using the Ff-nano dipstick assay and the range within which the signal depended on the amount of analyte, serial twofold dilutions of analyte (fibronectin) were assayed using this format. Since the dipstick assay signal was stronger in the unlabeled particle assay (**Figure 5B**) in comparison to the assay with FITC-labeled particles (**Figure 5C**), the former were used in this experiment.

**FIGURE 6:**
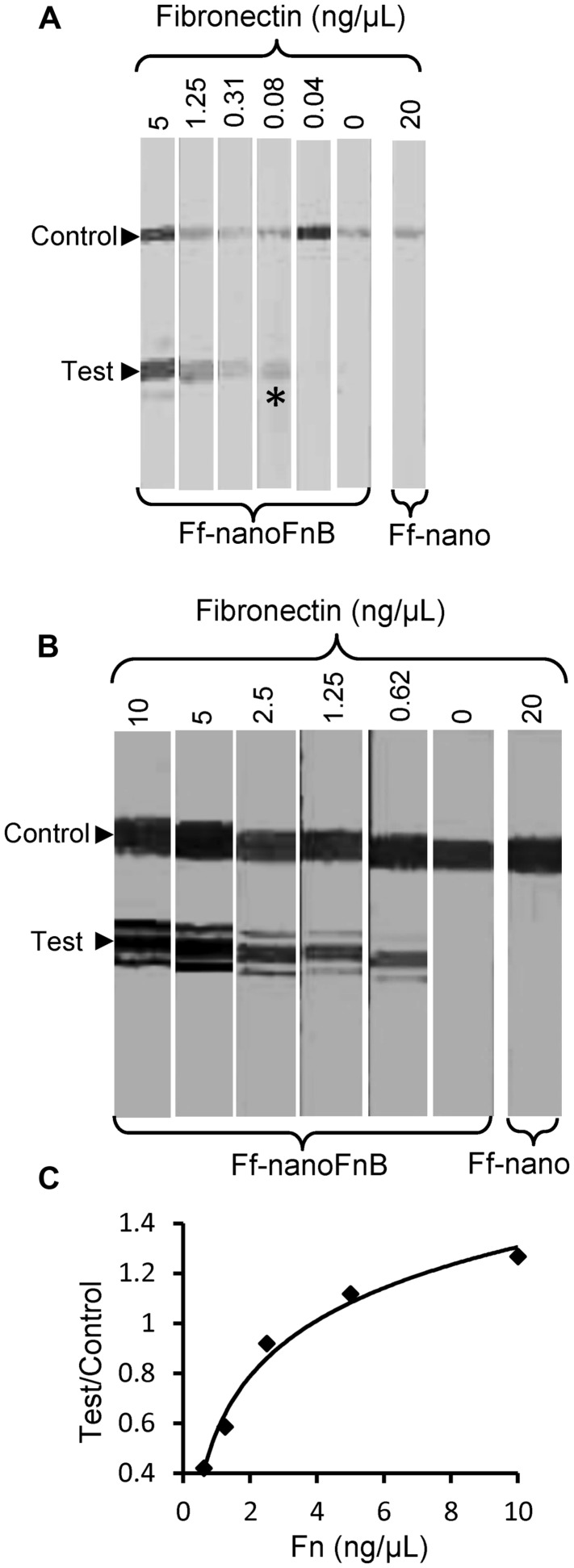
**Detection range of Fibronectin using Ff-nanoFnB.** Series of Fn (analyte) dilutions analyzed using Ff-nanoFnB-based dipsticks were used for determination of lower detection limit** (A)** and quantitative range **(B,C)**. Asterisk in **(A)** denotes the lowest Fn concentration at which the signal was detected on the test line. The graph in **(C)** corresponds to the Test/Control signal ratio vs. Fn concentration. Each assay contained Ff-nanoFnB particles (2 × 10^11^) mixed with Fn at the indicated final concentrations in a total volume of 50 μL. The assay was performed and the signal quantified as described in the Section “Materials and Methods.”

The lowest concentration of fibronectin at which a signal could be detected at the test line was 0.08 ng/μL, equivalent to 0.35 femtomoles/μL or 2 × 10^8^ molecules/μL, hence this is the sensitivity limit of the Ff-nanoFnB-based fibronectin detection dipstick assay (**Figure 6A**). The number of Ff-nano particles used in this assay was 2 × 10^11^, the highest number that did not show unspecific binding to nitrocellulose (data not shown).

Densitometric analysis of the signal showed that the ratio between the test and control lines was proportional to the log of Fn (analyte) concentration (**Figures 6B,C**) over a range of concentrations between 0.625 and 10 ng/μL, consistent with simple visual comparison of control band density. These are much lower concentrations of Fn than those measured in the serum of healthy individuals (259–400 ng/μL; Allard et al., 1986). A suitable serum dilution would therefore adjust the concentration to the quantitative detection range of the assay. Furthermore, variations of Fn concentration in disease fall within one order of magnitude relative to those of healthy individuals (Choate and Mosher, 1983; Cembrowski and Mosher, 1984; Weller et al., 1988; Honest et al., 2002; Mosher, 2006; Eissa et al., 2010).

## Discussion

### Ff-nano Production and Purification

Ff-derived functionalized nanoparticles reported to date are based on the long and thin filamentous template, whose hydrodynamic properties are not favorable for diffusion-based applications. From ethical and regulatory perspectives, the use of Ff-phage outside of the laboratory containment, in particular in home-use diagnostic devices or as carriers for tissue targeting of drugs, antigens, or diagnostic markers, is potentially controversial because they are recombinant viruses. Due to the propensity of Ff to infect Gram-negative bacteria via the ubiquitous TolQRA complex in the absence of their primary receptor, the F-pilus of *E. coli* (Russel et al., 1988; Heilpern and Waldor, 2000), they could potentially infect a wide range of Gram-negative bacteria. This includes a possibility of mobilization and horizontal gene transfer of antibiotic-resistance-encoding genes, present in most of the phage display vectors, among the gut or environmental bacteria. The Ff-derived particles (virions) containing phage display vectors have been shown to be taken up by mammalian cells in culture (Burg et al., 2002), hence there is a possibility that the encapsidated DNA permanently inserts into patient genomes.

To convert functionalized filamentous phage particles into non-replicating protein-DNA complexes, we have assembled functionalized short Ff-derived particles that contain 221-nt single-stranded DNA devoid of protein-coding sequences. Like Ff phage (f1, M13, and fd), genomes, the 221-nt ssDNA of the Ff-nano cannot integrate into *E. coli* chromosome as it lacks the necessary *cis* elements, such as genes for tRNA and/or sequences recognized by specific recombinases, that are used by some integrative filamentous phage to insert into bacterial chromosomes using the host recombinases (Rakonjac et al., 2011). Furthermore, given that it does not contain a negative (-) origin of replication, Ff-nano ssDNA cannot commence replication even if the specialized helper phage (containing *IRI* mutation in *gII*) coinfects the same cell. Therefore, Ff-nano improves the prospect of Ff filamentous phage applications outside of the laboratory. Given that these particles cannot replicate in any organism without extensive human intervention, they are considered not to be organsims in most legislatures regulating biological products, including the very strict New Zealand Hazardous Substances and New Organisms Act.

Furthermore, the length-to-diameter ratio of these particles is 15–20-fold smaller than that of the Ff particles derived from standard phagemid and phage vectors, improving the diffusion rate of the particles.

We have developed a production and purification pipeline for 50 nm particles by converting an Ff phage short-particle production system (Specthrie et al., 1992) into a high-efficiency system for producing functionalizable nanoparticles that we named Ff-nano. The increase in efficiency was achieved by using a high-copy-number plasmid containing the Ff-nano origin of replication and construction of helper phage Rnano3 and R408-3 that are also phage display vectors, containing cloning sites for construction of protein fusions to pIII.

In the Ff-nano purification, we introduced size-separation from the full-length helper phage by preparative agarose electrophoresis and electroelution. This strategy achieved excellent resolution, leading to a high enrichment of the Ff-nano particles over the full-length helper phage at a low cost and high recovery of the Ff-nano particles (31%). Overall, this production and purification system yields around 10^14^–10^15^ Ff-nano particles from 2 to 8 L of *E. coli* culture. The Ff-nano samples obtained, however, still contain full-length phage at an approximate frequency of 1 in 200,000 (5 × 10^-6^). Although the helper phage that we use do not contain antibiotic resistance genes, they still represent an undesired population that needs to be eliminated in order to obtain fully virus-free Ff-nano samples. Helper plasmids are expected to overcome this residual phage contamination in the Ff-nano preparations, as they do not contain a Ff origin of replication (Chasteen et al., 2006). A helper plasmid derived from helper phage VCSM13 (containing Km^r^ marker and p15A plasmid origin of replication, but not the *gII^IRI^* mutation) was constructed by deletion of the IG and tested for the Ff-nano production, however, it did not support the Ff-nano production to a detectable level, despite being efficient in supporting replication of standard phagemids that contain complete f1 origin of replication (Sattar, 2013). We have subsequently constructed a helper plasmid derived from Rnano3, which in preliminary analyses supported Ff-nano production (Jasna Rakonjac, unpublished). This helper plasmids is the next step toward the phage-free production of Ff-nano particles.

### Ff-nano Particle Stability

The Ff-nano were more resistant to heating in 1% (34 mM) ionic detergent SDS in comparison to the full-length phage. This property is solely due to the difference in phage length, as the assayed populations of full-length and Ff-nano particles were purified from the same *E. coli* culture and therefore had identical protein composition. Ff filamentous phage dissociation in the presence of SDS was investigated in detail by Stopar et al. (1998, 2002, 2003) using Electron Spin Resonance (ESR), circular dichroism and NMR. Solubilization of the major coat protein with detergent occurs when SDS displaces a pVIII subunit in the virion, replacing pVIII–pVIII interactions by pVIII-detergent interactions (Stopar et al., 1998). When a critical ratio of detergent to phage is reached, even a single pVIII subunit displacement across the filament triggers a cooperative dissociation of the virion (Ikehara et al., 1975; Stopar et al., 2002). In our experiment, the full-length virions were rapidly and completely disassembled after incubation in 1% SDS at 70°C for 5 min, which is the time point when over half of the Ff-nano particles in the sample remain intact (**Figure 3**). This length-dependent difference can be reconciled by the Ff-nano presenting a 17-fold smaller surface area for detergent interaction in comparison to the full-length phage, thereby decreasing the odds of steric imbalance caused by SDS–pVIII interactions per virion and resulting in much larger numbers of resistant particles under the same conditions. In addition, due to the short length of Ff-nano, the potential imperfections of pVIII packing that could occur in each particle due to mechanical bending and twisting of the filament are greatly reduced. Bending can be observed in the electron microscopic images of full-length phage (Gray et al., 1979; Rakonjac and Model, 1998), but not Ff-nano (**Figure 2A**; Supplementary Figure S5). The short length, preventing the bending of the filament may therefore confer additional stability to Ff-nano in comparison to full-length virions.

From the technological standpoint, the increased stability of Ff-nano particles may be beneficial in applications that involve high temperature in detergent-containing environment, or other harsh conditions. Given the higher resistance of Ff-nano to heating in 1% SDS at 70°C in comparison to complete sensitivity of the full-length phage, it is possible to develop conditions that will completely degrade the full-length phage in the purified Ff-nano preparation, while completely preserving the Ff-nano particles, thereby eliminating the full-length phage from the Ff-nano preparations. This approach will be possible for applications where the residue of SDS in the sample is not an issue, and where the Ff-nano particles display peptides that are resistant to denaturation by SDS.

### Ff-nano Functionalization and Use as a Detection Particle

To allow exploration of Ff-nano in biotechnological applications, we have converted the Ff-nano production system into a display system through insertion of MCS into *gIII* of the helper phage/vector, between the signal sequence and the mature portion of the protein. This system was then used to develop a dipstick fibronectin detection assay using Ff-nano displaying the fibronectin-binding domain from *S. pyogenes* (Ff-nanoFnB)*.* No unspecific binding to the analyte was detected using the Ff-nanoFnB detection particles, in contrast to residual non-specific signal observed using the full-length phage Rnano3FnB. The quantitative range of assay using Ff-nanoFnB detection particles was found to be between 0.625 and 10 ng/μL, with the logarithmic dependence between the Fn concentration of Fn and the ratio of the signal intensity between the test and control lines. This range appropriate for measuring the Fn concentration in human serum after a suitable dilution. Variations of Fn concentration can be used as indicators of several diseases, such as bladder cancer, liver damage, defibrination syndrome, arterial thrombosis, preterm birth, or ocular damage (Choate and Mosher, 1983; Cembrowski and Mosher, 1984; Weller et al., 1988; Honest et al., 2002; Mosher, 2006; Eissa et al., 2010). These variations fall within one order of magnitude, corresponding to the quantitative range of the Ff-nanoFnB-based dipstick assay. The Ff-nanoFnB can therefore be the basis for development of quantitative fibronectin assay.

In conclusion, this work describes a novel display system that functionalizes short Ff phage-derived nanorods. It further demonstrates one application for use in dipstick assays. Given a large range of publications describing applications where Ff virions are used, including the templates for assembly of inorganic structures, diagnostics, tissue templating, imaging, and drug targeting (Bar et al., 2008; Petrenko, 2008; Lee et al., 2009; Souza et al., 2010; Chung et al., 2011; Dang et al., 2013; Bernard and Francis, 2014; Oh et al., 2014), Ff-nano as short non-viral functionalized particles will likely find many diverse applications.

## Conflict of Interest Statement

The authors declare that the research was conducted in the absence of any commercial or financial relationships that could be construed as a potential conflict of interest.
