# Supplementary material for: Ff-nano, short functionalized nanorods derived from Ff (f1, fd, or M13) filamentous bacteriophage
Source: Front Microbiol. 2015 Apr 20;6:316. doi: 10.3389/fmicb.2015.00316 (PMC4403547; doi:10.3389/fmicb.2015.00316)
Supplement: Supplementary file 1 [file Presentation_1.PDF]

Supplementary Material

Ff-nano, Functionalized 50 nm x 6 nm Nanorods Derived from Ff Filamentous Bacteriophage

Sadia Sattar[1], Nicholas J. Bennett[1#], Wesley X. Wen[1], Jenness M. Guthrie[1,2], Len F. Blackwell[1,2], James F. Conway[3] and Jasna Rakonjac[1*]

[1]Institute of Fundamental Sciences, Massey University, Palmerston North, New Zealand
[2]EPAGSC, Palmerston North, New Zealand
[3]University of Pittsburgh School of Medicine, Pittsburgh, PA, USA
#Present address: Department of Medical Microbiology and Immunology, University of Alberta, Canada

*Correspondence: Associate Professor Jasna Rakonjac, Institute of Fundamental Sciences, Massey University, Private Bag 11-222, Palmerston North 4442, New Zealand
E-mail: j.rakonjac@massey.ac.nz

1. Supplementary Methods

1.1. Construction of recombinant phage and plasmids

To construct helper phage R777 (R408, *gVIII*^{am25}), the RF (double-stranded) DNA of R408 (Russel et al., 1986)and R676 (Feng et al., 1999) was cut with *BamHI* and *BsrGI*. The purified large *BamHI/BsrGI* fragment of R408 containing genes *gVI*, *gI*, *gXI*, *gIV*, *gII* and most of *gV* was ligated to the purified small *BamHI/BsrGI* fragment of R676 containing *gVII*, *gIX*, *gVIII* and of the 5' moiety of *gIII* (Suppl. Fig. 2) The ligation was transformed into electrocompetent cells of the *supD* strain K1030. To identify R408 *gVIII*^{am}, plaques obtained in the transformation plate were streaked onto the lawns of indicator srains. Recombinant phage clones that formed plaques on strain K1030, but not K561 transformed with pIV producing plasmid pPMR132, were consistent with the correct recombination product (replication in the absence of pIV due to a wild-type *gene IV*, but requiring the *supD* mutation due to the presence of the *gVIII*^{am25} mutation).

To construct the helper/vector Rnano3 (R777::MCS), a fragment containing MCS was amplified by overlap-extension PCR, using R777 as a template, flanking primers NJB6000 (5'-GTGCCTTCGT AGTGGCATTA-3') and NJB6004 (5'-ACATAAATCA ATATATGTGA GTGA-3') and overlapping primers containing multiple restrictions sites, NJB6001 (5'- GTTCCTTTCT ATTCTCACTC CGCGGCCCAG CCGGCCATGGG ATATCAGGCGGC-3') and NJB6003 (5'-CCATGGGATA TCAGGCGGCC GCTCCCGGGG GCGCTGAAAC TGTTGAAAGT TGTT-5'). The product was designed to amplify the flanking *gVIII*^{am25} allele and *gIII* sequences of R777, including the restriction sites *SnaB*I and *BamH*I. The product was cleaved with these two enzymes and inserted into the *SnaB*I-*BamH*I-cut phage R408 DNA (Suppl. Fig. 2). The correct recombinants were identified by the requirement of a *supD* mutation in the host for plaque formation as described above, except that strain K2092 served as a host to avoid restriction of the PCR-amplified insert by the rk$^+$, mk$^+$ strain K1030. The resulting helper phage-display vector was named Rnano3. It contains a multiple cloning site identical to the pHEN2 vector, including unique restriction sites for enzymes *Sfi*I, *Bgl*II, *Nco*I, *Eco*RV, *Xma*I, *and NotI*, as indicated in the figure.

Another helper/vector, named R408-3 that had wild-type gVIII was constructed. The MCS of R408-3 was identical to that of Rnano3; it was amplified using the same primers and strategy as that in Rnano3, except that R408 (instead of R777) served as a template (Suppl. Fig. 2). As in the Rnano3 construction, the MCS and the flanking phage genome sequences were cloned into *SnaB*I-*BamH*I-cut phage R408 and transformed into strain TG1.

To construct a recombinant helper/vector Rnano3FnB, displaying Fibronectin Binding Domain (FnB) from *Streptococcus pyogenes* (Rakonjac et al., 1995), the coding sequence for FnB was amplified from plasmid pDJ04 (Jankovic et al., 2007) using primers JR438 (5'-TCCCCCGCGG GAGGTCATGG ACCGATTGTC-3') and JR439 (5'-TCCCCCCGGG CTCGTTATCA AAGTGGAAGA AGC-3'). The JR438 and JR439 primers introduced a *Sac*II site and an *Xma*I site, respectively, at the ends of the product, which was cleaved with corresponding restriction enzymes and inserted into a *Sac*II-*Xma*I-cleaved Rnano3. The ligation mixture was transformed into strain K2092. The correct recombinants were verified by restriction analysis and sequencing.

The Ff-nano-producing plasmid pNJB7 was constructed by inserting the Ff-nano origin of replication, amplified using plasmid pLS7 (Specthrie et al., 1992) as a template and primers NJB26 (5'-

AGACGTTTTCCAGTTTGGAACAAG-3’) and NJB28 (5’-CCTATAAAAATAGGCGTATCACGAG-3’) into the vector pCR4-TOPO using the corresponding cloning kit (pCR4-TOPO blunt; Life Technologies, USA).

2. Supplementary Figures

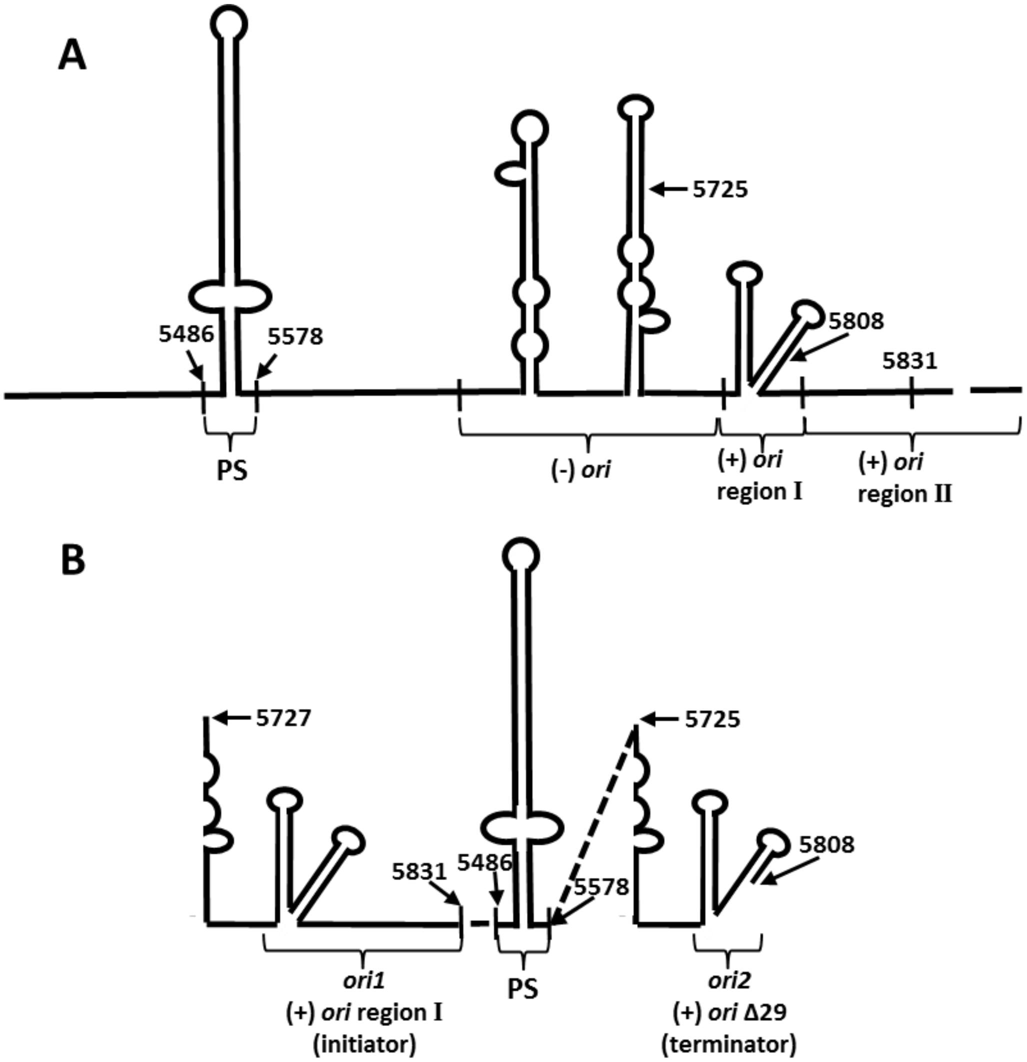

Supplementary figure 1. A. Ff wild-type intergenic sequence (IG) containing origin of replication. B. The microphage (Ff-nano) origin of replication in pNJB7. The sequence of ssDNA (+) strand is represented as a line folded in accordance to predicted secondary structure. Packaging signal (PS); (-) *ori*, negative strand origin of replication; (+) *ori* region I, the region I of the positive strand origin of replication, (+) *ori* region II, the region II of the positive strand origin of replication. *ori1*, region I of the (+) origin of replication (initiator); PS, packaging signal; *ori2*, positive origin deletion mutant (serves as a terminator) (Specthrie et al., 1992). Vertical lines (ticks) across the line representing ssDNA indicate borders of each of the indicated elements; arrows indicate the endpoints of DNA fragments used to construct the Ff-nano origin of replication. The numbers indicate the positions in the wild-type IG sequence (A) and the Ff-nano origin of replication (B) according to f1 genome sequence coordinates (Hill and Petersen, 1982). Dashed lines in (B) represent the sequences that link the f1-derived sequences; they are not according to the scale.

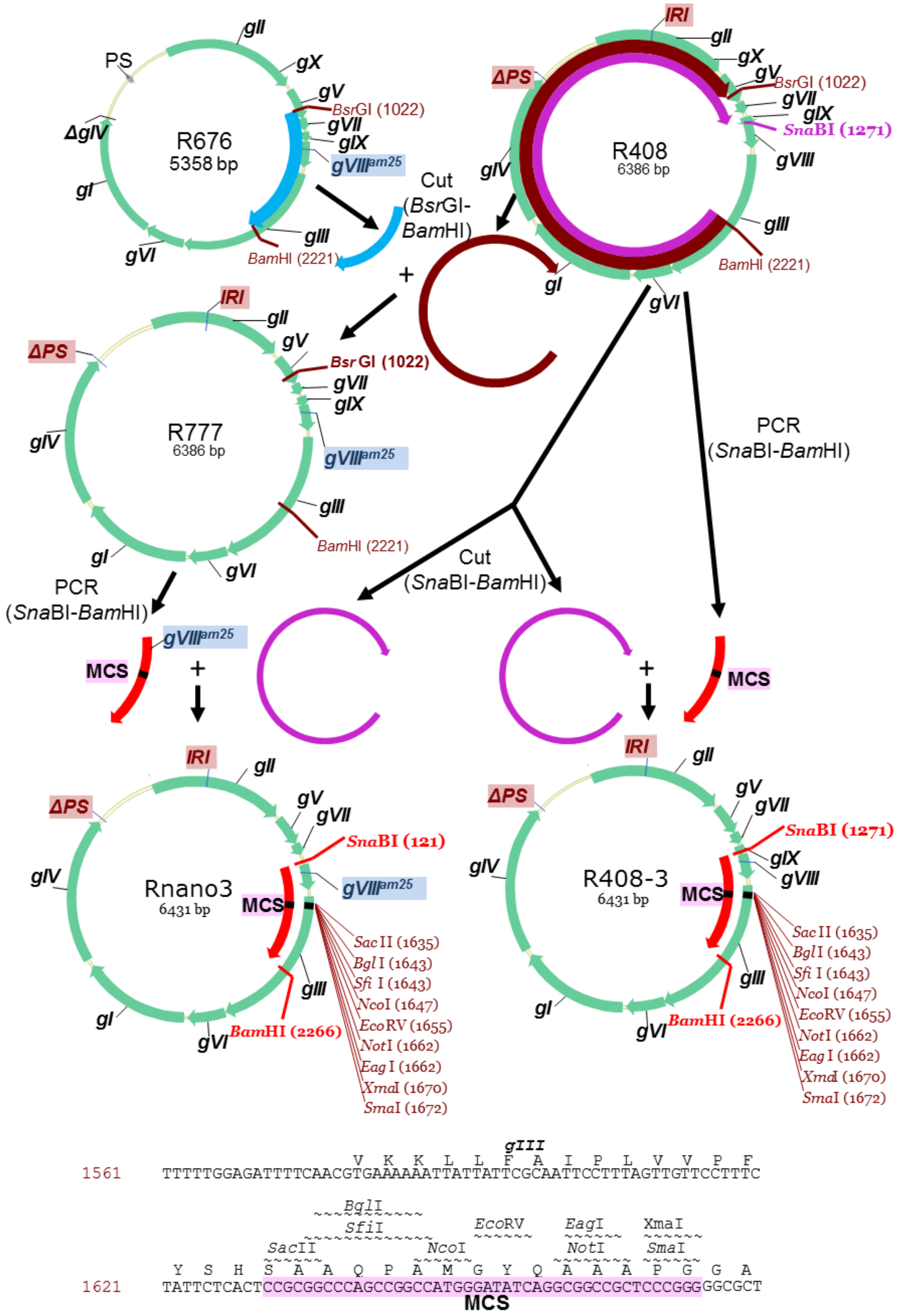

Supplementary figure 2. Construction of helper phage / display vectors Rnano3 and R408-3. R676, R408, R777, Rnano3 and R408-3 are all f1-derived helper phage. The MCS and surrounding sequence are shown on the bottom of the figure. Details of recombinant constructs in this work, including those shown in the figure, are given in Supplementary Methods section.

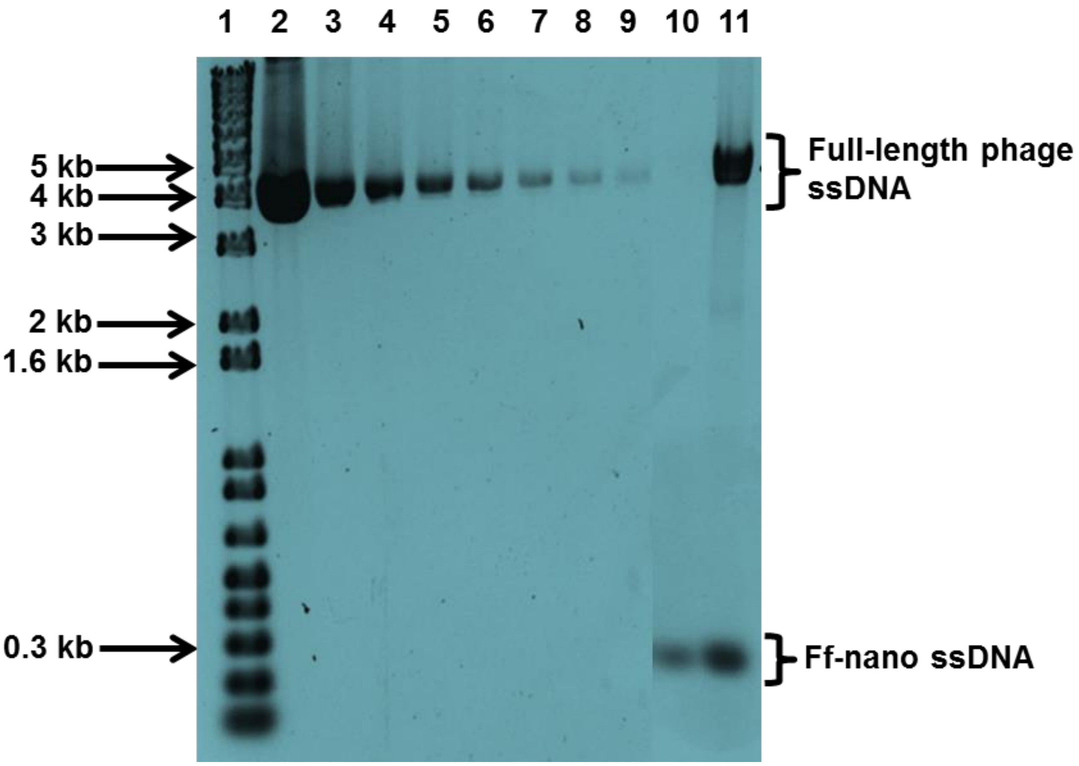

Supplementary figure 2. Quantification gel. Agarose gel electrophoresis of SDS-disassembled Ff-derived particles for quantification by densitometry. All samples were denatured by heating in SDS-containing buffer for 5 min at 100°C, to release ssDNA (please refer to Material and Methods section for details). The DNA was stained in ethidium bromide solution after electrophoresis. DNA bands were photographed using BioRad dark box and the band densities in the standard and test bands were determined using ImageQuant (Fuji). The amount of DNA and the copy number of the Ff-nano was calculated taking into account molecular weight difference relative to that of the full-length phage standard using Excel (Microsoft Office). Molecular weights were calculated based on the size and base composition as described (Rakonjac and Model, 1998). Lane 1, 1kb+ ladder (Life Technologies) that serves as a general migration marker; it is not suitable for size determination of phage circular ssDNA; Lanes 2-9, quantitation standard, two-fold serial dilutions of the f1 wild-type of known titre; 10, electropurified Ff-nano (5 µl out of 300 µl); 11, input into the preparative agarose electrophoresis Ff-nano-enriched lysate (5 µl out of 1 ml);

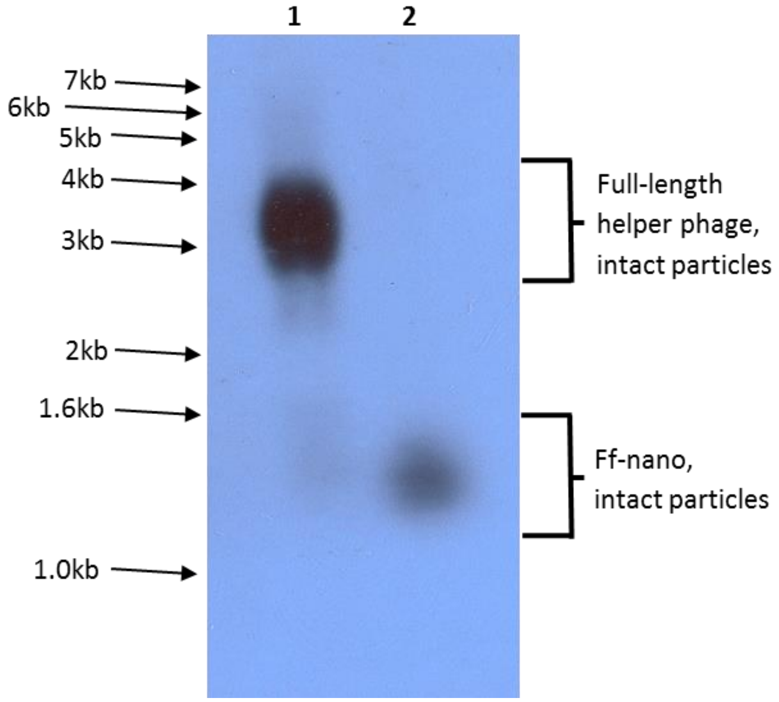

Supplementary figure 3. Separation of the intact full-length helper and Ff-nano by native agarose gel electrophoresis. Ff-derived particles from the supernatant of pNJB7-transformed K1030 cells infected with Rnano3 were precipitated by: 2.5% (low) PEG (lane 1). The remaining soluble fraction was precipitated by 15% (high) PEG, followed by further purification by preparative agarose electrophoresis (lane 2). Arrows indicate the position of the dsDNA fragments of the 1kb^{+} ladder (Invitrogen). Samples were mixed with the native agarose gel electrophoresis loading buffer and loaded on the gel (please refer to the Material and Methods section for details).

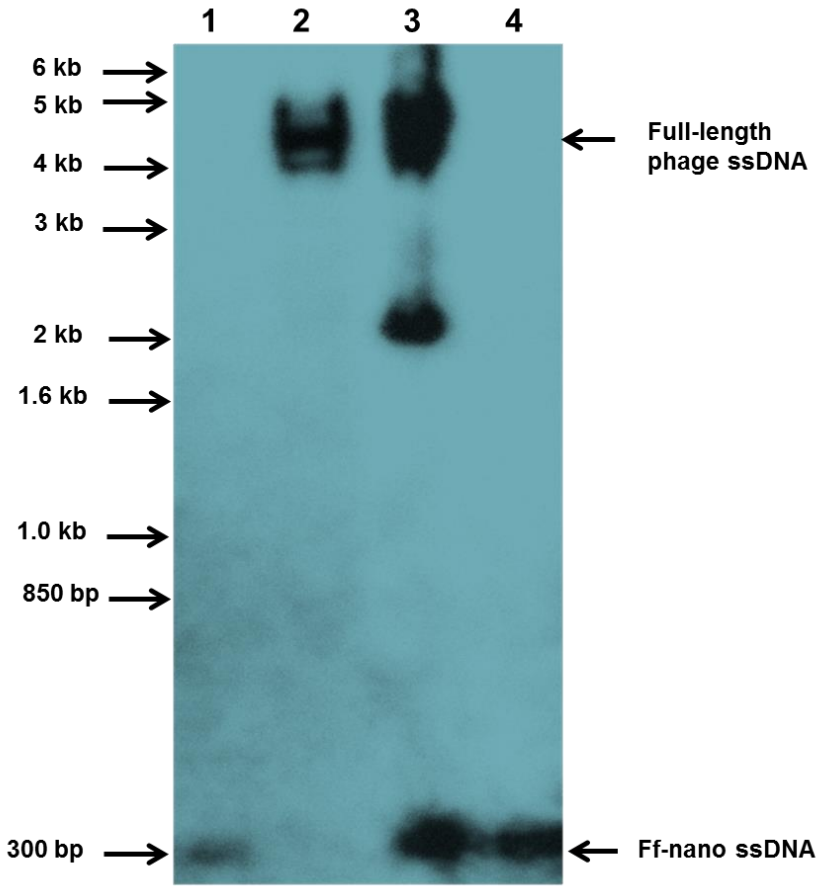

Supplementary figure 4. Comparison of Ff-nano production by Rnano3 and R408-3 as helper phage. Preparations enriched for the Ff-nano (lanes 1 and 4) and the full-length helper phage (lanes 2 and 3) were tested to determine the amount of Ff-nano produced using Rnano3 vs. R408-3. Virions from the high-PEG (Ff-nano enriched) fractions purified by preparative agarose electrophoresis or from the low-PEG (full-length phage enriched) fraction were disassembled by heating (5 min at 100 °C in the presence of 1% SDS) and the released ssDNA was separated by electrophoresis on an agarose gel. DNA was blotted onto the appropriate DNA-binding membrane and visualized by Southern blotting using a labelled PCR-generated probe corresponding to the origin of replication and packaging signal (the intergenic (IG) sequence using the ECL nucleic acids labelling and hybridization kit, GE Health, USA). The arrows indicate the position of 1 Kb Plus double-stranded linear DNA ladder bands (Life Technologies). This standard is not suitable for direct comparison of ssDNA size in nucleotides. It has been used only to measure the progression of electrophoresis and position of bands due to lack of an appropriate ssDNA marker.

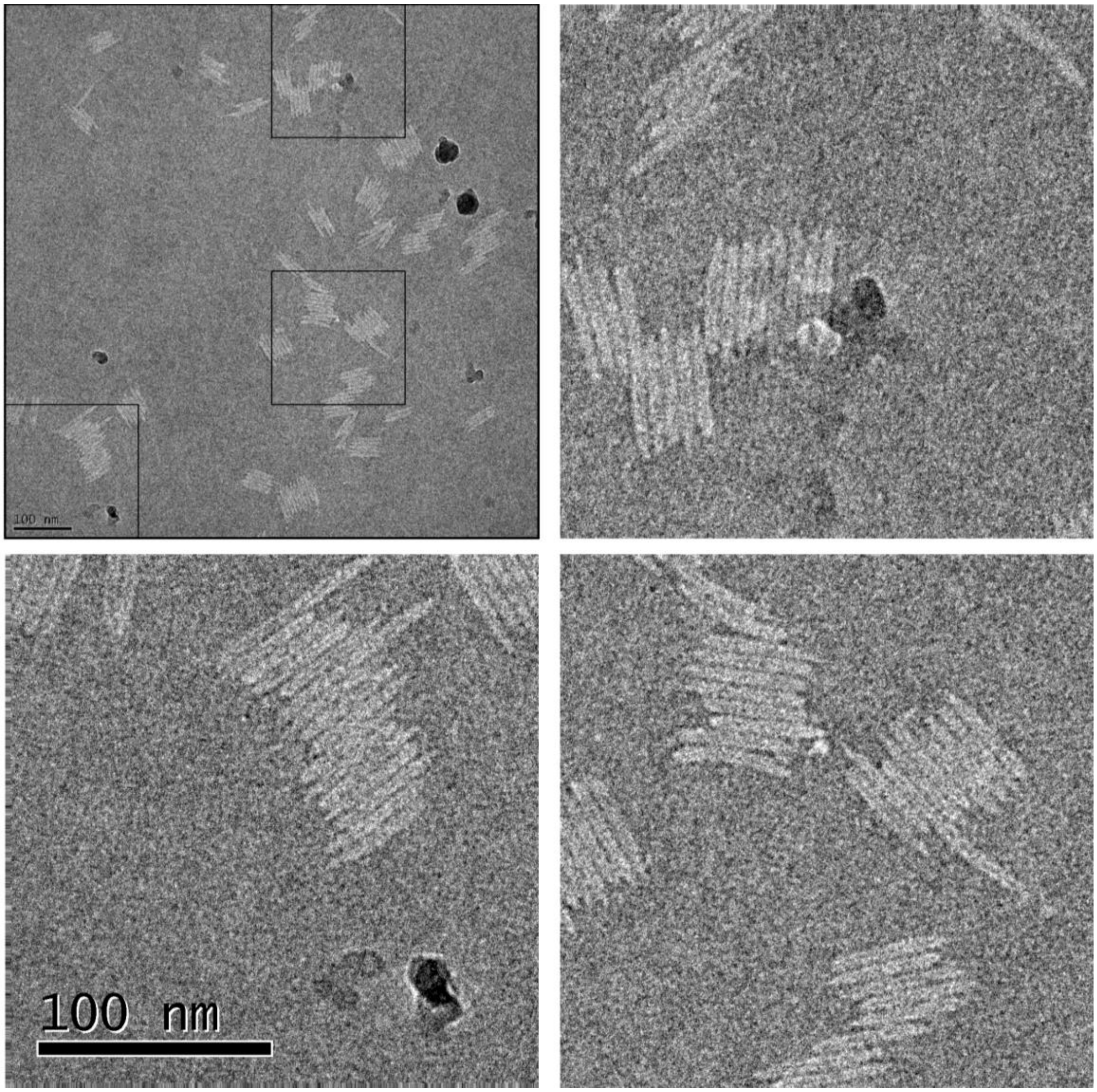

Supplementary figure 5: Cryo-negative electron micrograph image of purified R777-derived Ff-nano particles. This micrograph corresponds to Fig. 2A, except that it shows a wider vision field.

3. References

Feng, J.N., Model, P., and Russel, M. (1999). A trans-envelope protein complex needed for filamentous phage assembly and export. *Molecular Microbiology* 34, 745-755. .

Hill, D.F., and Petersen, G.B. (1982). Nucleotide sequence of bacteriophage f1 DNA. *J Virol* 44, 32-46.

Jankovic, D., Collett, M.A., Lubbers, M.W., and Rakonjac, J. (2007). Direct selection and phage display of a Gram-positive secretome. *Genome Biol* 8, R266.

Rakonjac, J., and Model, P. (1998). Roles of pIII in filamentous phage assembly. *J Mol Biol* 282, 25-41. doi: 10.1006/jmbi.1998.2006.

Rakonjac, J.V., Robbins, J.C., and Fischetti, V.A. (1995). DNA sequence of the serum opacity factor of group A streptococci: identification of a fibronectin-binding repeat domain. *Infect Immun* 63, 622-631.

Russel, M., Kidd, S., and Kelley, M.R. (1986). An improved filamentous helper phage for generating single-stranded plasmid DNA. *Gene* 45, 333-338.

Specthrie, L., Bullitt, E., Horiuchi, K., Model, P., Russel, M., and Makowski, L. (1992). Construction of a microphage variant of filamentous bacteriophage. *J Mol Biol* 228, 720-724.
